# Post-warm-up muscle temperature maintenance: blood flow contribution and external heating optimisation

**DOI:** 10.1007/s00421-015-3294-6

**Published:** 2015-11-21

**Authors:** Margherita Raccuglia, Alex Lloyd, Davide Filingeri, Steve H. Faulkner, Simon Hodder, George Havenith

**Affiliations:** Environmental Ergonomics Research Centre, Loughborough Design School, Loughborough University, Loughborough, Leicestershire LE11 3TU UK; Sport and Exercise Sciences Research Unit, University of Palermo, Palermo, Italy; School of Sport, Exercise and Health Sciences, Loughborough University, Loughborough, Leicestershire LE11 3TU UK

**Keywords:** Muscle temperature, Blood flow, Passive heating, Water perfused trousers, Occlusion

## Abstract

**Purpose:**

Passive muscle heating has been shown to reduce the drop in post-warm-up muscle temperature (*T*_m_) by about 25 % over 30 min, with concomitant sprint/power performance improvements. We sought to determine the role of leg blood flow in this cooling and whether optimising the heating procedure would further benefit post-warm-up *T*_m_ maintenance.

**Methods:**

Ten male cyclists completed 15-min sprint-based warm-up followed by 30 min recovery. *Vastus lateralisT*_m_ (*T*_mvl_) was measured at deep-, mid- and superficial-depths before and after the warm-up, and after the recovery period (POST-REC). During the recovery period, participants wore water-perfused trousers heated to 43 °C (WPT43) with either whole leg heating (WHOLE) or upper leg heating (UPPER), which was compared to heating with electrically heated trousers at 40 °C (ELEC40) and a non-heated control (CON). The blood flow cooling effect on *T*_mvl_ was studied comparing one leg with (BF) and without (NBF) blood flow.

**Results:**

Warm-up exercise significantly increased *T*_mvl_ by ~3 °C at all depths. After the recovery period, BF *T*_mvl_ was lower (~0.3 °C) than NBF *T*_mvl_ at all measured depths, with no difference between WHOLE versus UPPER. WPT43 reduced the post-warm-up drop in deep-*T*_mvl_ (−0.12 °C ± 0.3 °C) compared to ELEC40 (−1.08 ± 0.4 °C) and CON (−1.3 ± 0.3 °C), whereas mid- and superficial-*T*_mvl_ even increased by 0.15 ± 0.3 and 1.1 ± 1.1 °C, respectively.

**Conclusion:**

Thigh blood flow contributes to the post-warm-up *T*_mvl_ decline. Optimising the external heating procedure and increasing heating temperature of only 3 °C successfully maintained and even increased *T*_mvl_, demonstrating that heating temperature is the major determinant of post-warm-up *T*_mvl_ cooling in this application.

## Introduction

The importance of warming-up for subsequent short-duration/power-based exercise performance has been well documented (Asmussen and Boje 1945; Bergh and Ekblom 1979; Sargeant 1987; Hajoglou et al. 2005). Although prior exercise may induce psychological (Malareki 1954) and neuromuscular changes (Bishop 2003a, b) with a beneficial effect on performance, it has been suggested that the major contributing factor to post-warm-up performance improvement is the rise in muscle temperature (*T*_m_) (Bishop 2003a, b). Generally, *T*_m_ increases rapidly within the first 3–5 min of exercise, reaches a plateau after 10–20 min of activity and drops exponentially within 15–30 min after cessation of exercise (Saltin 1968; Faulkner et al. 2013b). It has been indicated that a recovery time of 15–20 min, between warm-up completion and the start of a sport event, allows for acid–base homeostasis (Bishop 2001), optimal balance between phosphocreatine restoration (Dawson et al. 1997) and muscle potentiation (Kilduff et al. 2013). However, it is not uncommon for athletes to experience a significantly longer recovery period (30–45 min) between active warm-up completion and their subsequent exercise performance (Mohr et al. 2004; Kilduff et al. 2013; West et al. 2013). For instance, cyclists perform a cycle-based warm-up approximately 30 min before the race, and this long delay has been shown to cause a significant reduction in *T*_m_, which has a detrimental effect on the following sprint performance (Faulkner et al. 2013a, b).

Our two previous studies have demonstrated that the use of heated trousers, during this period of inactivity, results in a significant attenuation in the *vastus lateralisT*_m_ (*T*_mvl_) drop (Faulkner et al. 2013a, b). The attenuated drop in T_mvl_ was consistently associated with a greater peak and mean power output (~9–11 and ~4 %, respectively) during 30-s maximal sprint, confirming the beneficial effect of an increased starting *T*_m_ on power-based performance. Nevertheless, despite the effectiveness of the heated trousers, a significant *T*_mvl_ drop of over 1.5 °C was still observed over the course of the recovery period. It has been suggested that leg blood flow could be a potential contributing factor to the decline in post-warm-up *T*_mvl_ (Faulkner et al. 2013a, b). In this regard, Kenny et al. (2003) suggested that post-exercise core temperature response is significantly influenced by conductive heat transfer from muscle to venous blood with subsequent convective transfer by the blood to the body core. In support Ducharme and Tikuisis (1994) showed that during immersion of the forearm and hand in water at 20 °C, heat exchange through convection between the blood and the forearm tissues accounted for 85 % of the total heat transferred. Conversely, during water immersion at 38 °C, the blood has the role of heat sink, transferring heat gained by the tissues from the environment to the rest of the body (Ducharme and Tikuisis 1994). According to the heat transfer analysis (Havenith 2001), the lower temperature of central blood, flowing into the warmer leg muscle tissue (post-warm-up), could have caused the previously observed reduction in *T*_mvl_, despite external heating. Furthermore, contributing to this may have been the effect of cooled blood returning from the lower leg and foot, subsequently cooling the muscle of the thigh as it returns to the body core. However to date, no research has examined the role of blood flow on *T*_mvl_ reduction, while using passive heating.

Another contributor to our previously observed reduction in *T*_mvl_ could be the microclimate created between the skin and trousers. It is possible that the previously adopted heating method did not provide enough heat to maintain *T*_mvl_. In fact, for safety reasons, the heating elements of the previously used heating trousers were designed not to exceed a temperature of 40 °C. Using electrical heating (Faulkner et al. 2013a, b), a safety margin for the heater temperature is required to avoid skin burns (45 °C) as changes to the heating element insulation (e.g. sitting on a chair) will change the temperature achieved by the heaters.

In order to investigate the aforementioned mechanisms (i.e. blood flow and optimal microclimate temperature), in the current study lower limb arterial and venous blood circulation was restricted in a single leg during the post-warm-up recovery. As such, the contribution of blood flow, as a cooling source, was studied by comparing *T*_mvl_ between the occluded and the perfused leg. Furthermore, to investigate the role of cooled blood returning to the thigh from the unheated lower leg (Faulkner et al. 2013a, b) (via venous return), post-recovery *T*_mvl_ was observed in two different conditions: whole leg heated (WHOLE) and upper leg heated only (UPPER).

Lastly, using circulating liquid heating, local overheating (encountered with the electrically heated trousers) does not occur; therefore, the application of a liquid heating system allowed us to increase the external temperature from ~40 to 43 °C (without any skin damage), and thus, the study of the role of an increased heating temperature (microclimate) on *T*_mvl_ declines.

The occlusion of the leg blood flow, required to answer the research question posed, causes muscle ischemia and a numb leg and therefore precludes this experimental design from incorporating a performance test. The impact of *T*_m_ on performance has been repeatedly demonstrated (Asmussen and Boje 1945; Bergh and Ekblom 1979; Sargeant 1987; Hajoglou et al. 2005; Faulkner et al. 2013a, b), and a positive dose–response relation for post-warm-up *T*_m_ and performance is assumed (more detail on this will be presented in discussion). Therefore, a performance test was not considered necessary to answer the research question posed.

In summary, the purpose of this study was twofold. First, we aimed to investigate the role of central (core) and peripheral (lower leg) blood flow on *T*_mvl_ during 30 min of passive recovery. We hypothesised that *T*_mvl_ would be (1) lower in the perfused leg compared to the occluded leg, due to the heat loss to arterial blood coming from the core and (2) lower in the upper leg heating only *versus* whole leg heating, due to the higher heat loss to the cooled venous blood returning from the unheated lower leg and foot. Secondly, we aimed to examine the effect of heating temperature on *T*_mvl_ during the course of the passive recovery period. We hypothesised that the application of an optimised (higher) heating temperature will further reduce the previously observed drop in *T*_mvl_ over the course of the recovery period. To assess the latter, *T*_mvl_ results achieved by water-perfused trousers (43 °C heating temperature) were compared to *T*_mvl_ data obtained from our previous heating method (up to 40 °C) and control group (no passive heating), in which the exercise protocol adopted was identical to that of the current study.

## Methods

### Participants

Ten male cyclists volunteered to participate in this investigation (age 22.0 ± 0.8 years; height 180.0 ± 2.3 cm; body mass 76.5 ± 5.4 kg; mean ± SD). With respect to the training status of the participants, all of them performed at least three cycle-based training sessions per week as well as other regular physical activity, such as running and weight lifting. They completed a general health-screening questionnaire and were all non-smokers, free from injury and from any medication. All subjects were informed verbally of the objectives and procedures of the study before giving written informed consent. In the 24 h prior to each trial, participants were asked to refrain from caffeine, alcohol ingestion and any strenuous exercise. They were also asked to keep a record of their food intake and replicate this prior to the subsequent visit. The protocol and procedures involved were approved by the Loughborough University Ethical Advisory Committee. The study was conducted within the confines of the World Medical Association Declaration of Helsinki for medical research using human participants.

For the comparison of different heating temperatures/methods, a between-group comparison was made to the data presented by Faulkner et al. (2013a, b). Eleven male cyclists (age = 24.7 ± 4.2 years, height = 1.82 ± 0.72 m, body mass = 77.9 ± 9.8 kg; not significantly different from the present experimental group) with the same level of weekly activity were exposed to the exact same warm-up and recovery protocol.

### Study overview

Participants visited the laboratory on three occasions. Prior to the main experimental trials, participants were familiarised with exercise testing and the general measurement procedures used in the present study. On the two remaining visits, participants performed the experimental tests. On each occasion, after 30 min of stabilisation period, participants completed a 15-min cycle sprint warm-up, followed by 30 min of passive recovery. In both experimental trials, during the passive recovery, arterial and venous blood flow of one leg was occluded (NBF), whereas the other leg was used to observe the effect of blood flow on thigh muscle temperature decline (BF). The two visits differed in the surface area over which heat was applied; in one visit, upper and lower leg were warmed up (WHOLE), while in the other visit only the upper leg was kept warm (UPPER). The trials were completed in a balanced order, separated by a minimum of 72 h. In order to make the current results comparable to that from previous studies (Faulkner et al. 2013a, b), all experiments were performed in a temperature and humidity-controlled environment maintained at 17.7 ± 0.3 °C and 54.0 ± 3.0 % relative humidity, which Faulkner et al. (2013a, b) considered a range relevant to athletic events in a Western European climates (originally aiming at the London Olympics).

### Experimental protocol

On the two experimental sessions, participants entered the controlled climatic room environment and were instrumented with skin, rectal and heart rate measurements systems. Participants remained lying on a bench for 30 min, wearing shorts and t-shirt, to allow time for muscle, core and skin temperatures to stabilise. After the stabilisation period, baseline measures for thigh muscle temperature (*T*_mvl_) at three different depths for both legs, quadriceps skin temperature (*T*_sk-quad_), gastrocnemius skin temperature (*T*_sk-gas_), core temperature (*T*_c_) and heart rate (HR) were taken. These parameters were recorded every 1 min during the experimental trials, apart from *T*_mvl_, which was recorded after the stabilisation period (PRE), on completion of the warm-up exercise (POST-WUP) and immediately after the recovery period (POST-REC). After the stabilisation period, participants mounted a cycle ergometer and performed the standardised active warm-up protocol which consisted of 5-min cycling at an external power output of 100 W, followed by five 10-s maximal sprints (10-s Wingate test), each sprint separated by 1 min 55 s of cycling at 75 W (Faulkner et al. 2013a, b). Participants were asked to maintain a cadence of 85 rpm, until the start of each sprint. The maximal sprints were performed at a frictional load equivalent to 10 % body mass. On completion of the warm-up, participants dismounted the ergometer, laid supine on the bench (this took ~30 s), where POST-WUP *T*_mvl_ was measured. Following this, a blood pressure cuff was immediately applied proximally around the right thigh to abolish/minimise leg blood flow, and the participant donned the water-perfused trousers (WPT). The mean inflation pressure was 140 mmHg. To ensure that the inflation pressure was supra-systolic, individual blood pressure was measured before the recovery period. During all conditions, the WPT were connected to a controlled water bath (45.0 ± 0.1 °C) and reached a temperature of 43.0 ± 0.5 °C. This temperature was selected after extensive pilot testing, as it was the maximum temperature for which no signs of superficial skin injury or pain were present. Once donned, the participants remained in the environmental chamber in a supine position for 30 min. At the end of the 30-min recovery, the water-perfused trousers were removed and a POST-REC *T*_mvl_ measurement was taken. After this measurement, the blood pressure cuff was removed and the participant remained in the lying position until the stabilisation reached baseline parameters.

In addition, to assess the effectiveness of the optimised heating procedure (WPT43) in maintaining post-warm-up *T*_mvl_, POST-REC *T*_mvl_ data were compared with *T*_mvl_ data from two of our previous studies, where participants donned electrically heated trousers heated at 40 °C (ELEC40) and non-heated tracksuit bottoms (CON) during 30 min of passive recovery subsequent to the same warm-up exercise protocol used in this study. The current group of subjects (WPT43) and those from our previous studies (ELEC40 and CON) were homogenous in terms of gender, age, anthropometric characteristics and fitness level (Faulkner et al. 2013a, b).

### Measurements

#### Core and skin temperature

To monitor changes in core temperature, rectal temperature (*T*_c_) was recorded throughout the experimentation. Participants inserted a rectal thermistor (Grant Instrument Ltd, Cambridge, UK) 10 cm beyond the anal sphincter. Local skin temperature over both quadriceps (*T*_sk-quad_) and gastrocnemius (*T*_sk-gas_) was measured using calibrated thermistors (Grant Instrument Ltd, Cambridge, UK), two for each region, placed at the following sites on the legs: muscle belly of rectus femoris and the widest diameter of the gastrocnemius. Temperatures were recorded at 1-min intervals using a Grant Squirrel 2010 data logger (Grant Instrument Ltd, Cambridge, UK).

#### Muscle temperature

Muscle temperature of the *vastus lateralis* of each leg was measured using a solid needle probe with an inbuilt thermocouple (Ellab, Copenhagen, Denmark). The following muscle temperature measurement procedure was developed after extensive pilot studies. The needle was inserted 1/3 distal to the line that runs from the insertion of the vastus lateralis to the iliac crest. The insertion of the vastus lateralis was estimated at 1/4 distal of the distance between the midpoint of the base of patella and the midpoint of popliteal fossa.

The insertion depth of the needle probe was corrected for adipose tissue. The adipose tissue thickness was calculated using a skinfold calliper over the site of the insertion. The average skinfold thickness of participants was 6 ± 0.8 mm.

The needle was inserted at 25, 15 and 5 mm depths beyond the muscle fascia according to

$${\text{Depth of insertion }}\;{ = }\;{\text{required depth }}\left( {\text{mm}} \right) \;{ + }\; \frac{1}{2} {\text{local skinfold thickness }}\left( {\text{mm}} \right).$$

Thus, in this experiment, the average depth of insertion (including adipose calculation) was 28 mm (deep-*T*_mvl_), 18 mm (mid-*T*_mvl_) and 8 mm (superficial-*T*_mvl_). To measure *T*_mvl_ at each depth, the needle probe was inserted at the deepest depth of 28 mm, then withdrawn to 18 mm, and finally withdrawn again to 8 mm depth. The needle was inserted at the same location for each measurement period (PRE, POST-WUP and POST-REC). To allow for stabilisation of the measurement, the needle was inserted for ~3 s at each depth. The temperature was measured using a hand-held digital thermometer (Ellab, Copenhagen, Denmark).

#### Heart rate

Heart rate was recorded every 5 min using a wireless heart rate monitor (RS800, Polar, Kempele, Finland).

#### Water-perfused trousers

During the 30-min recovery period, muscle passive heating was achieved using bespoke high-density water-perfused trousers (WPT; Med-Eng System Inc., Pembroke, Canada) consisting of 2.5-mm-internal diameter medical grade PVC tubing, sewn in a pair of trousers. The tubing covered the following individually perfused segments: buttocks, front and back upper legs, and lower legs. The WPT were composed of spandex mesh fabric. The garment has a series of snap fasteners on each side running from the groin region to the ankles to ensure a tight fit. Additionally, to allow an adjustment of the tightness, two strips were integrated around each leg, as well as to minimise heat transfer between the hot water running through the suit and the cooler air temperature of the environmental chamber, a cover garment was placed over the WPT. The water flow was always kept at a fixed value of 250 cm^3^/min (total 1000 cm^3^/min) for each of the four zones, which allows maximum skin and muscle warming at each segment. The WPT were connected to a heating system, consisting of a temperature-controlled water bath and powered water pump. In the UPPER sessions, the tubing covering the lower segments of both legs was disconnected from the system.

#### Blood pressure cuff

Blood flow was restricted using arterial occlusion of the leg. An adjustable blood pressure cuff (Hokanson, Bellevue, WA 98005 USA) was applied around the proximal portion of the thigh, as close as possible to the groin region. For each participant, the same (right) leg was used for the blood flow intervention in each condition (WHOLE versus UPPER*)*. The blood pressure cuff was connected by a PVC tube to a custom-made compressor able to inflate the blood cuff with a supra-systolic pressure (140 mmHg). This was measured before the recovery period and shown to ensure full blood flow restriction.

#### Thermal discomfort

Thermal discomfort of the legs and whole body was recorded at 5-min intervals throughout each of the experimental trials by using a 5-point scale (‘do you find this’: 0 = comfortable; 1 = slightly uncomfortable; 2 = uncomfortable; 3 = very comfortable; 4 = extremely uncomfortable) (ISO 1995).

### Statistics

A preliminary analysis was conducted to ensure that there were no violations to the assumption of normality of distribution. Data were analysed using 3-way repeated measures ANOVA with a Bonferroni correction. The effect of the independent variables (blood flow, heating procedure and muscle depth) was observed over the time on *T*_mvl_ (PRE, POST-WUP and POST-REC), *T*_c_, *T*_sk-quad_, *T*_sk-gas_ and HR (PRE, 5WUP, 10WUP, 15WUP, 5REC, 10REC, 15REC, 20REC, 25REC and 30REC). In addition to the *p* value, the effect size index (partial Eta squared, $\eta_{p}^{2}$) was included to assess the proportion of variance in *T*_mvl_ in response to the heating procedure (WHOLE versus UPPER). Effect size index is interpreted as 0.10 small effect, 0.30 medium effect and 0.50 large effect (Cohen 1988). One-way between-group ANOVA was conducted to assess the effect of the passive heating (CON; ELEC40; WPT43) on *T*_mvl_. Where significant effects were identified, post hoc multiple comparison tests with Fisher LST correction were conducted. The accepted level of significance was set a priori to *p* ≤ 0.05. Data are presented as mean ± SD. The software SPSS I20 was used for the statistical analysis.

## Results

### Muscle temperature

**Fig. 1:**
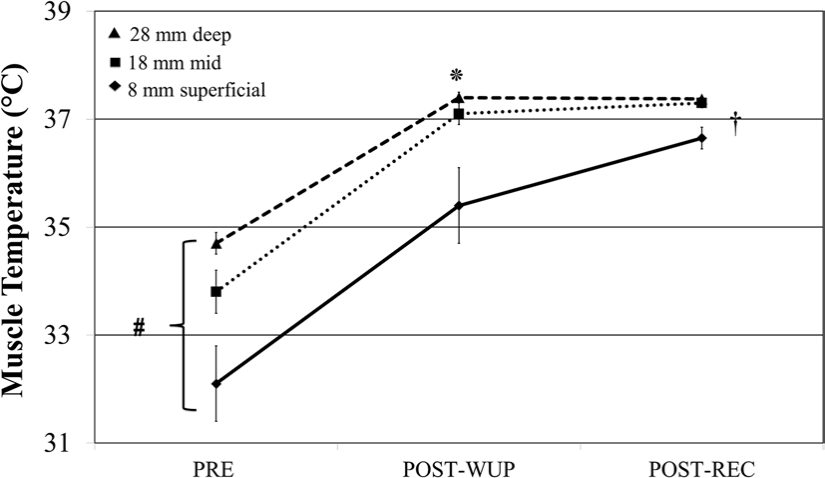
Muscle temperature change from baseline (PRE) to post-warm-up (POST-WUP) and post-recovery (POST-REC) at 28, 18 and 8 mm depths. ^#^Significant effect of depth on muscle temperature at both PRE and POST-WUP time points (*p* < 0.05). *Significant effect of warm-up on muscle temperature at all depths (p < 0.001). ^**†**^Significant effect of passive warm-up on muscle temperature at all depth (*p* < 0.01). PRE and POST-WUP data are presented as the average of both legs. POST-REC muscle temperature is presented as the average of the perfused leg (BF) data collapsed over heating procedure WHOLE versus UPPER

Resting (PRE) *T*_mvl_ at each depth was not significantly different between legs (right versus left), blood flow conditions (BF versus NBF) or heating procedure (WHOLE versus UPPER). However, PRE *T*_mvl_ did vary significantly (*p* < 0.05) across all measured depths (28, 18 and 8 mm): deep-*T*_mvl_ (34.7 ± 0.8 °C) was significantly different from mid-*T*_mvl_ (33.8 ± 1.4 °C); deep- and mid- *T*_mvl_ were significantly different from superficial-*T*_mvl_ (32.1 ± 2.4 °C). After the warm-up (POST-WUP), *T*_mvl_ increased by 2.7 ± 0.6, 3.2 ± 1.2 and 3.3 ± 1.3 °C above resting values for 28, 18 and 8 mm, respectively (Fig. 1), narrowing the temperature gradient between depths (*p* < 0.001). POST-WUP *T*_mvl_ was unaffected by blood flow conditions (BF *versus* NBF) and heating procedure (WHOLE versus UPPER).

**Fig. 2:**
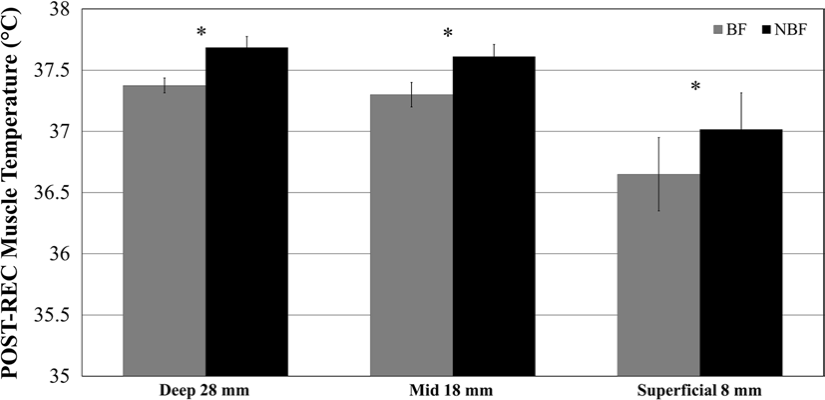
Thigh muscle temperature post 30-min recovery period (POST-REC) collapsed for heating procedure (WHOLE versus UPPER) at deep (28 mm)-, mid (18 mm)- and superficial-*T*_mvl_ (8 mm) depths. *Blood flow significantly decreased (*p* < 0.05) muscle temperature in BF (circulated leg) compared to NBF (occluded leg) at all measured depths

POST-REC *T*_mvl_ was not significantly different (*p* > 0.05; $\eta_{p}^{2}$ < 0.10) between heating procedure (deep-*T*_mvl_ WHOLE 37.5 ± 0.3 °C versus deep-*T*_mvl_ UPPER 37.5 ± 0.2 °C; mid- *T*_mvl_ WHOLE 37.5 ± 0.4 °C versus mid- *T*_mvl_ UPPER 37.4 ± 0.5 °C; superficial-*T*_mvl_ WHOLE 36.6 ± 1.1 °C versus superficial-*T*_mvl_ UPPER 37.0 ± 0.8 °C), and there was no interaction between heating procedure and blood flow. Therefore, *T*_mvl_ data were collapsed for heating procedure (WHOLE versus UPPER) and are presented in the two conditions BF and NBF. POST-REC *T*_mvl_ was lower in BF versus NBF: 0.31 ± 0.3 °C for deep-*T*_mvl_; 0.31 ± 0.4 °C for mid-*T*_mvl_ and 0.36 ± 0.4 °C for superficial *T*_mvl_ (all *p* < 0.05, Fig. 2).

**Fig. 3:**
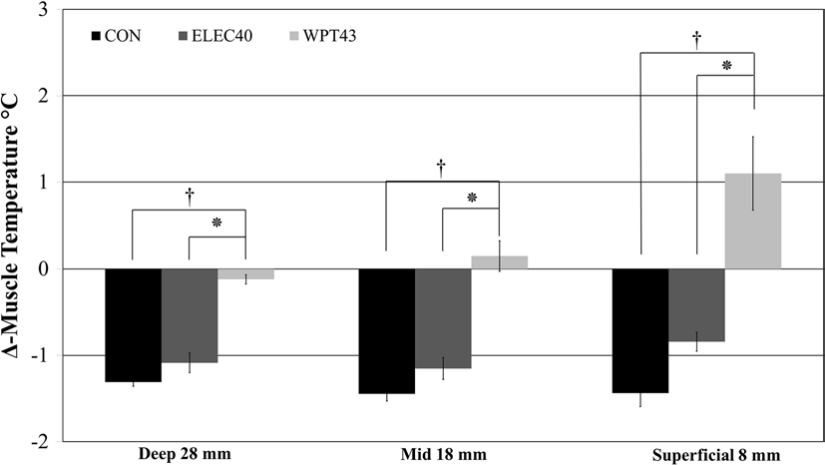
Normalised muscle temperature (difference from post-warm-up) recorded after 30 min of passive recovery following a standardised sprint cycling warm-up in CON (tracksuit bottoms), ELEC40 (electrically heated trousers at 40 °C) and WPT43 (trousers perfused with water at 43 °C) at deep (28 mm)-, mid (18 mm)- and superficial-*T*_mvl_ (8 mm) depths. ^†^Significant effect (*p* < 0.001) of WPT43 on post-warm-up *T*_mvl_ compared to CON. *Significant difference (*p* < 0.001) between the two heating procedures: ELEC40 and WPT43

Post-recovery period *T*_mvl_ values obtained from our previous electrical heating method (ELEC40) and control group (non-heated tracksuit bottoms; CON) were compared to POST-REC BF T_mvl_ data achieved with the current optimised heating procedure (WPT43) (Fig. 3). In WPT43, the reduction in *T*_mvl_ during recovery was significantly attenuated or even eliminated compared to ELEC40 and CON (*p* < 0.001). Deep-T_mvl_ decreased by 0.12 ± 0.3, 1.08 ± 0.4 and 1.3 ± 0.3 °C in WPT43, ELEC40 and CON condition, respectively. Mid- *T*_mvl_ increased by 0.15 ± 0.3 °C in WPT43, whereas it was reduced by 1.15 ± 0.2 and 1.44 ± 0.2 °C in ELEC40 and CON conditions, respectively. Finally, superficial-*T*_mvl_ increased by 1.1 ± 1.1 °C in WPT43 condition and dropped by 0.8 ± 0.5 and 1.4 ± 0.3 °C in ELEC40 and CONT conditions, respectively.

### Heart rate and core temperature

**Fig. 4:**
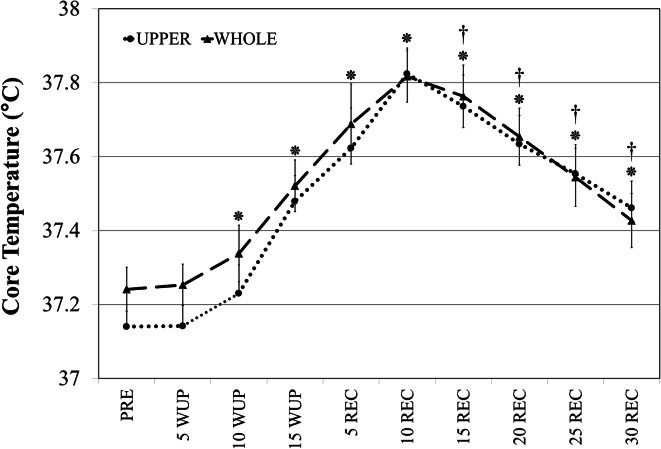
Rectal temperature recorded every 5 min over the course of the trial in both WHOLE and UPPER conditions: after 30 min of stabilisation (PRE), during 15 min of active warm-up (5WUP; 10WUP; 15WUP), and during 30 min of passive recovery (5REC; 10REC; 15REC; 20REC; 25REC; 30REC). *Significant increase (*p* < 0.05) in core temperature from PRE. ^**†**^Significant decrease (*p* < 0.05) in core temperature from 15REC

There was no effect of blood flow conditions (BF *versus* NBF) and heating procedure (WHOLE versus UPPER) on both HR (Table 1) and *T*_c_ (Fig. 4) during the trials. In both WHOLE and UPPER conditions *T*_c_ increased significantly (*p* < 0.05) at time points 10WUP, 15WUP, 5REC, 10REC, 15REC, 20REC, 25REC and 30 REC compared to PRE and 5WUP. At time point 10REC, *T*_c_ reaches the peak and significantly decreases (*p* < 0.05) at time points 15REC, 20REC, 25REC and 30 REC compared to 10REC.

**Table 1:** Quadriceps skin temperature (*T*_sk-quad_), Gastrocnemius skin temperature (*T*_sk gas_) for whole leg heating (WHOLE) and upper leg heating only (UPPER), core temperature (*T*_c_) and heart rate (HR), measured after 30-mins recovery period and each 5 mins during active warm-up (WUP) and passive recovery period (REC)

	PRE	5WUP	10WUP	15WUP	5REC	10REC	15REC	20REC	25REC	30REC
*T*_sk-quad_ (°C)	30.9 ± 0.7	30.6 ± 0.6	30.7 ± 0.8	30.6 ± 1.1	30.7 ± 1.1	32.2 ± 1.9	35.6 ± 1.8	37.3 ± 1.5	37.9 ± 1.3	38.4 ± 1.3
*T*_sk-gas WHOLE_ (°C)	30.4 ± 1.1	29.4 ± 1.5	29.2 ± 1.7	29.0 ± 2.1	29.4 ± 2.0	31.7 ± 1.6	33.7 ± 1.9	36.0 ± 0.9	36.9 ± 0.9	37.9 ± 0.7
*T*_sk-gas UPPER_ (°C)	30.4 ± 0.8	29.6 ± 0.9	29.2 ± 1.3	29.1 ± 1.6	29.5 ± 1.8	30.5 ± 1.7	31.9 ± 1.5	32.9 ± 1.3	33.4 ± 1.2	34.1 ± 1.1
*T*_c_ (°C)	37.2 ± 0.2	37.2 ± 0.2	37.3 ± 0.2	37.5 ± 0.3	37.6 ± 0.3	37.8 ± 0.3	37.7 ± 0.3	37.6 ± 0.3	37.5 ± 0.3	37.4 ± 0.l
HR (bpm)	71 ± 10	110 ± 16	153 ± 15	152 ± 22	107 ± 12	108 ± 12	106 ± 12	105 ± 12	106 ± 12	105 ± 12

All data presented as mean ± SD

### Quadriceps skin temperature

Baseline *T*_sk-quad_ was 30.9 ± 0.7 °C and not different between legs (right versus left), blood flow conditions (BF versus NBF) and heating procedure (WHOLE versus UPPER). During the 15 min warm-up *T*_sk-quad_ did not increase in response to the exercise. During the recovery period (REC), *T*_sk-quad_ increased continuously in response to the passive heating, and at 30 min into REC, it was significantly elevated (38.4 ± 1 °C) above PRE (*p* < 0.05) (Table 1) but not different between conditions (BF versus NBF; WHOLE versus UPPER).

### Gastrocnemius skin temperature

After the stabilisation period (PRE), *T*_sk-gas_ was 30.4 ± 0.9 °C with no difference between blood flow conditions (BF versus NBF) and heating procedure (WHOLE versus UPPER). During the warm-up exercise, *T*_sk-gas_ decreased to 29.0 ± 1.8 °C (*p* < 0.05). During the recovery period (REC), *T*_sk-gas_ increased continuously in response to the passive heating (*p* < 0.05). At 10 min into REC (10 REC), *T*_sk-gas_ was significantly different (*p* < 0.001) between heating procedure (WHOLE *versus* UPPER) but not between blood flow (BF *versus* NBF). At the end of the recovery period (30 REC), WHOLE *T*_sk-gas_ was 37.9 ± 0.7 °C, whereas UPPER *T*_sk-gas_ was 34.1 ± 1 °C (Table 1).

### Whole-body thermal comfort

Thermal discomfort of the whole body was not affected by blood flow conditions (BF *versus* NBF) and heating procedure (WHOLE versus UPPER). Over time whole body thermal discomfort varied significantly (*p* < 0.01). After 30 min of stabilisation period (PRE), thermal comfort was 0 (“comfortable”), during the warm-up exercise increased to 1 (“slightly uncomfortable”) and over the recovery period was 0.7 (between “comfortable” and “slightly uncomfortable”).

### Legs thermal discomfort

A similar trend was shown by legs’ thermal discomfort; it was different (*p* < 0.01) over time, and after the stabilisation period (PRE) the score was 0 (“comfortable”) and reached the value of 0.6, between “comfortable” and “slightly uncomfortable”, during the warm-up exercise. During the recovery period (REC), heating procedure (WHOLE versus UPPER) did not affect legs thermal discomfort. During REC BF, thermal discomfort increased (*p* < 0.05) to 1.6, between “slightly uncomfortable” and “uncomfortable”, whereas in NBF, it was 1.8 and not different from thermal discomfort achieved during the warm-up.

## Discussion

This study demonstrates that blood perfusion of the thigh muscles contributes to the cooling of *T*_mvl_ during 30 min of passive recovery, following an active warm-up, even when the leg is passively heated. Conversely, heating the lower leg, in addition to the upper leg, did not affect post-recovery *T*_mvl_. Additionally, the use of an optimised heating procedure, consisting of trousers perfused with water at 43 °C, instead of electric heating at 40 °C, can increase mid- and superficial-*T*_mvl_ rather than just reducing the drop and considerably reduce the post-warm-up drop in deep-*T*_mvl_. Thus, we accept our hypotheses that both the central (core) blood flow and the heating temperature contribute to the reduction in post-warm up *T*_mvl_.

### Muscle temperature

Confirming previous results (Saltin et al. 1968; Kenny et al. 2003), baseline *T*_mvl_ was different across all measured depths: 34.7, 33.8 and 32.1 °C, for deep-, mid- and superficial-*T*_mvl_, respectively. This is in line with the work of Saltin et al. (1968) who found that at rest deep-*T*_mvl_ is around 33–34 °C. In the current study, warm-up exercise increased *T*_mvl_ by 2.7, 3.2 and 3.3 °C for deep-, mid- and superficial-depths, respectively, reducing the temperature gradient across the three depths. Additionally, after 30 min of passive recovery, *T*_mvl_ of the perfused leg was lower compared to *T*_mvl_ of the occluded leg at all measured depths (~0.3 °C for deep- and mid- *T*_mvl_; ~0.4 °C for superficial-*T*_mvl_). In the perfused leg, the optimised external temperature resulted in a small decrease in deep-*T*_mvl_ and in an increase in mid- and superficial-*T*_mvl_ (0.15 and 1.1 °C, respectively) compared to post-warm-up values.

### The role of external temperature on post-warm-up muscle temperature

A central aim of this study was to examine the role of passive heating temperature on the decline in post-warm-up *T*_mvl_ and whether a relatively small increase in heating temperature (40–43 °C) would provide a measurable benefit. The heating temperature of the previous trousers was set to a maximum of 40 °C. Since the trousers were electrically heated, it was not possible to control the local heating temperature precisely and further increases in the heating element’s temperature could have caused local overheating and skin burns. During piloting for this study, we observed that an external temperature of 40 °C resulted in a mean leg *T*_sk_ of ~37 °C, which was not enough to maintain *T*_mvl_ achieved with the active warm-up (Faulkner et al. 2013a, b). The use of circulated liquid heating allowed the safe application of an optimised, higher heating temperature (43 °C) which resulted in a higher quadriceps *T*_sk_ (~38 °C; Table 1), thus minimising the temperature gradient between *T*_sk_ and *T*_mvl_. The optimisation of the heating procedure lead to a substantial reduction of the deep-*T*_mvl_ drop compared to our previous studies (Faulkner et al. 2013a, b), where we reported a ~1 °C decline, and to increases rather than decreases in mid and superficial-*T*_mvl_. The present data indicate that microclimate temperature is a major contributing factor in post-warm-up *T*_mvl_ decline and that even relatively small changes in heating temperature have an impact. Furthermore, the greater increase in superficial-*T*_mvl_ indicates, as to be expected, that the superficial tissues are most affected by microclimate and ambient temperature, which may be the most important for cycling sprint performance (Faulkner et al. 2013b).

### The contribution of blood flow on post-warm-up muscle temperature

The higher *T*_mvl_ in the occluded condition confirms the cooling effect of blood flow. This could be by arterial blood from the core, venous blood from the lower leg, or both. The fact that rectal temperature remains higher than muscle temperature throughout the recovery period would suggest that arterial blood could not be responsible. However, rectal temperature is often higher (~0.3 °C) than arterial blood temperature (Taylor et al. 2014), and oesophageal temperature, better representing arterial temperature, decreases faster than rectal temperature post-exercise (Gagnon et al. 2010). Therefore, it is likely that arterial blood temperature entering the thigh was lower than muscle temperature, during, at least, part of the recovery period; this would support a role of arterial blood flow on post-warm-up muscle temperature reduction, as observed in the present study. However, since in this study arterial blood temperature was not measured, the idea that it may have been lower than muscle temperature remains an assumption.

The temperature difference in *T*_mvl_ between perfused and occluded leg was relatively small (~0.3 to ~0.4 °C for different depths) which could be due to the high heating temperature used in this study. In fact, it is possible that the optimised passive heating could also reduce the drop rate in central and peripheral blood temperature post-warm-up, reducing the temperature gradient between blood and muscle and therefore narrowing the temperature difference between the perfused and occluded leg. Nevertheless, the effect of blood flow on *T*_mvl_ is still evident, though it would likely have been more pronounced with the electric heating system at 40 °C (Faulkner et al. 2013a, b).

We hypothesised that peripheral blood, together with central blood flow, could contribute to the post-warm-up *T*_mvl_ decline. To verify our hypothesis, post-recovery *T*_mvl_ was observed in two different conditions: WHOLE and UPPER. However, while we expected a lower thigh *T*_mvl_ in the UPPER condition compared to WHOLE condition, thigh muscle temperature was not different between heating interventions. It is possible that the high blood temperature (resulting from the higher heating temperature used) flowing from the thigh to the lower leg, increased the temperature of the lower leg tissue in the UPPER condition. In support of this, skin temperature of the lower leg and foot showed a significant increase of 4 °C during the recovery period even if heat was not directly applied, whereas in the whole leg heated condition (WHOLE), it increased by 7 °C. Therefore, these results indicate that, when using an optimised heating method, heating the calf in addition to the thigh does not further increase quadriceps muscle temperature, compared to heating the thigh only. However, for sports where calf muscles are relevant, lower leg heating may still provide a performance benefit due to increased local *T*_m_.

Blood flow restriction coupled with the use of passive heating resulted in a *T*_mvl_ increase at all depths. In this study, blood flow occlusion was used as means to understand the mechanistic effect of circulating blood flow on post-warm-up *T*_mvl_ decline, rather than a proposed tool to maintain *T*_mvl_ during a period of inactivity. In fact, 30 min of blood flow occlusion is expected to have a detrimental effect on performance, as the restriction of circulation would not allow the wash-out of metabolites accumulated in the leg during exercise, causing muscle discomfort and peripheral fatigue (Bigland-Ritchie et al. 1986; Gandevia et al. 1996; Amann et al. 2013).

As the study of blood flow precluded doing a performance test, it needs to be considered whether the observed improved maintenance of *T*_m_ would result a in further sprint–power performance enhancement above those observed in Faulkner et al.’s (2013a, b) muscle heating studies. Optimal *T*_m_ is thought to be ~39 °C, with an upper threshold of 43 °C, above which there are impairments to muscle function (Åstrand and Rodahl 1986; McRae and Esrick 1993). Although the relationship between improved sprint performance and increased *T*_m_ may not follow complete linearity, Zochowski et al. (2007) and West et al. (2013) showed that shorter recovery periods (down to 10 and 20 min from 45 min) between warm-up and race, with concomitant smaller drops in *T*_m_, did result in a greater improvement in the following sprint performance. The latter strongly indicate that the smaller the post-warm-up *T*_m_ drop, the better the subsequent sprint/power performance, thus supporting that the optimised heating method used here has a strong potential of further performance enhancement.

## Conclusion

This study has demonstrated that to maintain post-warm-up muscle temperature, an increase of the leg heating temperature from 40 to 43 °C is sufficient to virtually abolish the deep thigh muscle temperature drop. Additionally, a 3 °C higher external temperature can even increase superficial muscle temperatures during a 30-min recovery period. Further, it was observed that blood perfusing the thigh during the period of inactivity following an active warm-up is one of the factors responsible for the earlier observed reductions in thigh muscle temperature. Heating the lower leg in addition to the upper leg however did not further improve thigh muscle temperature. Nevertheless, in an applied sports performance setting, additional performance benefits from lower leg heating on the lower leg muscles may be evident. Given the cooling effect of blood flow observed, and according to the local heat balance, lower leg heating could also improve upper leg *T*_mvl_ in less optimised heating systems as used in earlier studies from our laboratory.

Given that the water-perfused heating system in its current form may not be practical for use in the field, further product development needs to be considered by the sporting goods industry, now that the present study has demonstrated that optimisation of the heating system can provide further gains in muscle temperature maintenance.

## Abbreviations

BF: Blood flow
CON: Control (non-heated)
ELEC40: Electrically heated trousers to 40 °C
NBF: No blood flow
*T*_c_: Core temperature
*T*_mvl_: Vastus lateralis muscle temperature
*T*_sk-gas_: Gastrocnemius skin temperature
*T*_sk-quad_: Quadriceps skin temperature
UPPER: Upper leg heating
WHOLE: Whole leg heating
WPT43: Water perfused trousers heated to 43 °C

## References

[CR1] Amann M, Venturelli M, Ives SJ, McDaniel J, Layec G, Rossman MJ, McDaniel J, Layec G, Rossman MJ, Richardson RS (2013). Peripheral fatigue limits endurance exercise via a sensory feedback-mediated reduction in spinal motoneuronal output. J Appl Physiol.

[CR2] Asmussen E, Boje O (1945). Body temperature and capacity for work. Acta Physiol Scand.

[CR3] Åstrand PO, Rodahl K (1986). Textbook of work physiology. Physiological bases of exercise.

[CR4] Bergh U, Ekblom B (1979). Influence of muscle temperature on maximal muscle strength and power output in human skeletal muscles. Acta Physiol Scand.

[CR5] Bigland-Ritchie BR, Dawson NJ, Johansson RS, Lippold OC (1986). Reflex origin for the slowing of motoneurone firing rates in fatigue of human voluntary contractions. J Physiol.

[CR6] Bishop D (2003). Warm up I: potential mechanisms and the effects of passive warm up on exercise performance. Sports Med.

[CR7] Bishop D (2003). Warm up II: performance changes following active warm up and how to structure the warm up. Sports Med.

[CR8] Bishop D, Bonetti D, Dawson B (2001). The effect of three different warm up intensities on sprint kayak performance in trained athletes. Med Sci Sports Exerc.

[CR9] Cohen J (1988). Statistical power analysis for the behavioral sciences.

[CR10] Dawson B, Goodman C, Lawrence S (1997). Muscle phosphocreatine repletion following single and repeated short sprint efforts. Scand J Med Sci Sports.

[CR11] Ducharme MB, Tikuisis P (1994). Role of blood as heat source or sink in human limbs during local cooling and heating. J Appl Physiol.

[CR12] Faulkner SH, Ferguson RA, Gerrett N, Hupperets M, Hodder SG, Havenith G (2013). Reducing muscle temperature drop after warm-up improves sprint cycling performance. Med Sci Sport Exerc.

[CR13] Faulkner SH, Ferguson RA, Hodder SG, Havenith G (2013). External muscle heating during warm-up does not provide added performance benefit above external heating in the recovery period alone. Eur J Appl Physiol.

[CR14] Gagnon D, Lemire BB, Jay O, Kenny GP (2010). Aural canal, esophageal, and rectal temperatures during exertional heat stress and the subsequent recovery period. J Athl Train.

[CR15] Gandevia SC, Allen GM, Butler JE, Taylor JL (1996). Supraspinal factors in human muscle fatigue: evidence for suboptimal output from the motor cortex. J Physiol.

[CR16] Hajoglou A, Foster C, De Koning JJ, Lucia A, Kernozek TW, Porcari JP (2005). Effect of warm-up on cycle time trial performance. Med Sci Sport Exerc.

[CR17] Havenith G (2001). Individualized model of human thermoregulation for the simulation of heat stress response. J Appl Physiol.

[CR18] ISO 10051 FS (1995). Ergonomics of the thermal environment-assessment of the influence of the thermal environment using subjective judgement scales.

[CR19] Kenny GP, Reardon FD, Zaleski W, Reardon ML, Haman F, Ducharme MB (2003). Muscle temperature transients before, during, and after exercise measured using an intramuscular multisensor probe. J Appl Physiol.

[CR20] Kilduff LP, West DJ, Williams N, Cook CJ (2013). The influence of passive heat maintenance on lower body power output and repeated sprint performance in professional rugby league players. J Sci Med Sport.

[CR21] Malareki I (1954). Investigation of physiological justification of so-called ‘warming-up’. Acta Physiol Pol.

[CR22] McRae DA, Esrick MA (1993). Changes in electrical impedance of skeletal muscle measured during hyperthermia. Int J Hyperthermia.

[CR23] Mohr M, Krustrup P, Nybo L, Nielsen JJ, Bangsbo J (2004). Muscle temperature and sprint performance during soccer matches–beneficial effect of re-warm-up at half-time. Scand J Med Sci Sports.

[CR27] Saltin B, Gagge AP, Stolwijk JA (1968). Muscle temperature during submaximal exercise in man. J Appl Physiol.

[CR30] Sargeant AJ (1987). Effect of muscle temperature on leg extension force and short-term power output in humans. Eur J Appl Physiol Occup Physiol.

[CR31] Taylor NAS, Tipton MJ, Kenny GP (2014). Considerations for the measurement of core, skin and mean body temperatures. J Therm Biol.

[CR32] West DJ, Dietzig BM, Bracken RM, Cunningham DJ, Crewther BT, Cook CJ (2013). Influence of post-warm-up recovery time on swim performance in international swimmers. J Sci Med Sport.

[CR33] Zochowski T, Johnson E, Sleivert GG (2007). Effects of varying post-warm-up recovery time on 200-m time-trial swim performance. Int J Sports Physiol Perform.

